# Optimization of orange juice formulation through using lactose‐hydrolyzed permeate by RSM methodology

**DOI:** 10.1002/fsn3.1774

**Published:** 2020-07-08

**Authors:** Azita Nemati, Mohammad Alizadeh‐ Khaledabad, Zahra Ghasempour

**Affiliations:** ^1^ Department of Food Science and Technology Faculty of Agricultural Engineering Sari Agricultural Sciences and Natural Resources University Sari Iran; ^2^ Department of Food Science and Technology Urmia University Urmia Iran; ^3^ Department of Food Science and Technology Tabriz University of Medical Sciences Tabriz Iran

**Keywords:** lactose hydrolysis, optimization, orange juice, permeate

## Abstract

Permeate is the by‐product of the process of ultrafiltration in a kind of cheese making process in which a semipermeable membrane filters the liquid. It mainly contains 4.5%–4.8% lactose and 0.44%–0.47% mineral salts which make it a safe disposal issue. This study was conducted to use permeate and its lactose as an alternative to sugar, and to use these useful permeate compounds in an optimized orange juice formulation. Milk permeate, as a waste disposal of dairy companies, was applied in lactose hydrolyzed form as the cost effective sugar and water substitution in production of orange juice. The RSM optimization method was applied for formulating beverage mixture. The heated and nonheated permeate samples were incubated with β‐glycosidase enzyme in three thermal ranges (35, 40, and 45°C), 3 time intervals (60,150, and 240 min), and 3 enzyme levels (0%, 0.1%, and 0.2%). The degree of hydrolysis was determined by MilkoScan analyzer. In the next step, optimization of orange juice was accomplished with a mixture of sugar (10%–40%) and hydrolyzed permeate (10%–40%) with specific Brix through RSM statistical design. The physicochemical properties and sensory evaluation were measured during 8 weeks of storage. At the first stage of the study, the heated sample with 0.1% enzyme density, which was incubated for 150 min at 40°C, was yielded the best result. At the second stage, which was the juice production and evaluation, the statistical analysis showed increasing trend of pH and sugar content, but density and vitamin C showed a decreasing trend during storage time (*p* < .05). The optimal condition was obtained in taking 35% permeate and 41 days of storage in which the values of formalin, vitamin C, and sensory tests were in the highest levels.

## Graphical abstract

Optimum conditions for permeate hydrolysis by β‐galactosidase were obtained. Sample with 0.1% enzyme content during 150 min incubation at 40°C gave the best results. Hydrolyzed permeate substituted as a part of orange juice sugar and Brix.

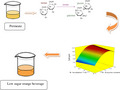

## 1 INTRODUCTION

Milk permeate, which is the by‐product of ultrafiltration process of milk, has been regarded as a waste product. It is a rich source of lactose and nutraceutical ingredients such as the essential amino acids and soluble proteins, immunoglobulin, lactoferrin, lactoperoxidase, vitamins, growth factors, and minerals (Dutra Rosolen, Gennari, Volpato, & Volken de Souza, 2015). As a waste disposal of dairy companies, it is important to use this by‐product in food processing (Chavan, Shraddha, Kumar, & Nalawade, 2015).

However, the lactose presence in permeate and dairy products limits the consumption of these products by lactose‐intolerant individuals, as a result of the reduction in the β‐galactosidase enzyme levels in intestinal walls. Therefore, food industries have been requesting to develop low‐lactose or lactose‐free products. Thus, lactose enzymatic hydrolysis with β‐galactosidase enzyme, leading to glucose and galactose monosaccharides production, emerges as a key biotechnological process with application in the dairy industry. The lactose hydrolysis improves digestibility and its functional properties, and it also has more relative sweetness factor than the lactose. The β‐galactosidase enzyme is derived from a variety of sources, such as microorganisms, plants, and animals, and is used in the food industry for lactose hydrolysis in milk and whey. This enzyme can be used in free or stabilized forms (Beucler, Drake, & Allen, 2005).

Bayoumi, Mohamed, and El‐Sheikh (2011) have conducted a study on chocolate milk prepared by permeate and found that the use of permeate in the composition of the chocolate milk reduced the amount of the required sugar, which was more favorable for economic and health reasons. Dhamsaniya and Varshney (2013) studied on a banana beverage made of milk permeate, and they found that the best fit was the ratio of 15 ml of banana juice, 3 ml of herbal extract, 8 g of sugar, and 77 ml of milk permeate. Singh, Khemariya, and Rai (2014) studied on lemon juice from hydrolyzed whey and reported that the percentage of sugar required for the beverage had been decreased. Salama, Salem, and Yousef (2014) have studied on the production of new milk permeate beverage, which had been fortified with dried leaves of *Moringaoleifera* (DLMO), as an innovative beverage. In Rahimi, Kalbasi Ashtari, Labbafi, Longnecker, and Khodayian (2015) researches, optimization of new grapefruit beverage formulation using milk permeate was accomplished.

Nowadays, due to nutritional and health concerns, consumers, especially young people and women, are looking for high‐value nutritional drinks that are called healthy drinks. During the first 9 months of 2004, 44 new products were introduced in Europe. As a result, many beverages, called healthy beverages, entered the market. It is claimed that most of these drinks are healthy (low calories, no preservatives, artificial colors, etc.). Drinks with positive ions such as sodium and potassium, as well as high‐value nutritional drinks for consumption as a full meal, have been also developed for the athletes. The fruit drinks are among the most common drinks, one of which is dairy‐based fruit drinks. The proteins of dairy product and its compounds such as whey and permeate are greatly used in beverages, which contain proteins and lactose and are often used in sports drinks as sources of protein and energy (Baljeet, Ritika, & Sativa, 2013; Gad, Emam, Mohamed, & Sayd, 2013; Hattem, Abouel‐Einin, & Mehanna, 2011; Pareek, Gupta, & Sengar, 2014; Prashanth, Jayaprakash, Soumyashree, & Madhusudhan, 2018; Rizk, 2016).

This study intended to use lactose‐hydrolyzed permeate to replace a large part of water and also the sugar in an optimized orange drink formulation to produce a marketable, natural, and healthy product with high nutritional qualities in addition to decrease the wasted disposal and the environment pollution. The freezing point was considered as an indicator of lactose hydrolysis. Introducing lactose‐hydrolyzed permeate in orange juice formulation was studied through RSM optimization method. The produced orange juices were evaluated through physicochemical analysis including formalin index, reducing sugar amount, ascorbic acid measurement, and sensory evaluation.

## 2 MATERIALS AND METHODS

### 2.1 Materials

The permeate was obtained from the ultrafiltration of whole milk in the Pegah Dairy Co. The orange concentrate was prepared from Noosh Co. and stored in refrigerated condition (−20°C) before its utilization. The β‐galactosidase of the Christian Hansen was used to hydrolyze permeate samples.

### 2.2 Permeate enzymatic hydrolysis

**FIGURE 1:**
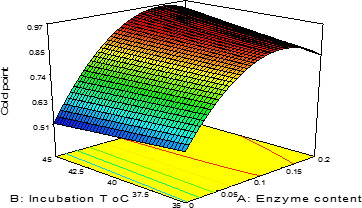
Effect of incubation T °C and enzyme content on cold point

The heated and nonheated permeate samples were incubated and hydrolyzed by β‐galactosidase in different thermal range, time intervals, and enzyme levels according to experimental design (Table 1). The degree of hydrolysis was analyzed by using the MilkoScan instrument. The results showed that the heated sample with 0.1% enzyme content during 150 min and in 40°C gives the best results (Figure 1).

**Table 1:** Different treatments of permeate based on RSM experimental design

Run	Enzyme content (%)	Incubation temperature(^o^C)	Incubation time (min)	Heat treatment
1	0	45	60	No
2	0.2	45	60	No
3	0	45	240	No
4	0.2	35	240	Yes
5	0.2	45	240	No
6	0	45	60	Yes
7	0.1	40	150	No
8	0.2	35	60	Yes
9	0.1	40	150	Yes
10	0.1	40	150	Yes
11	0.2	35	240	No
12	0	35	240	No
13	0	35	60	No
14	0	45	240	Yes
15	0	35	60	Yes
16	0.2	45	60	Yes
17	0	35	240	Yes
18	0.2	45	240	Yes
19	0.1	40	150	Yes
20	0.1	40	150	No
21	0.2	35	60	No

To prepare this selected sample for the next stage, the permeate was heated at 85°C for 5 min in a hot water bath. Then, it was filtered using filter paper. Once the permeate temperature reached 35°C, 0.1% of the enzyme was injected and then the permeate sample was well shaken to disperse the enzyme (Beucler et al., 2005). The sample was incubated in optimum conditions (40°C for 150 min). The results of permeate analysis are shown in (Table 2).

**Table 2:** Permeate analysis

pH	Brix	TS	Turbidity	Fat	Protein
6.56	5.63	5.6	22.2	0	0.2

### 2.3 Beverage preparation

The beverage samples were prepared by using orange concentrate and sugar mixture (10, 14.5, 25, 35, and 40) and hydrolyzed permeate (10, 14.5, 25, 35, and 40), which provided 50% of the total Brix of the beverage. The samples were placed in the beaker on a magnetic stirrer and they were mixed up together and then were pasteurized at 90°C for 5 min (Singh et al., 2014). In the next step, the cold juice was poured into sterile capped bottles and stored at a refrigerated temperature. The physicochemical properties of the samples including pH, reducing sugar, vitamin C, and sensory properties were measured during 60 days storage.

## 3 MEASUREMENT OF PHYSICOCHEMICAL INDICES

The acidity was measured in terms of lactic acid based on titration and the pH was measured by a digital laboratory pH meter (WTW537) with glass electrode. The protein was measured by the Kjeldahl analysis method, the density was measured by pycnometer, and the Gerber method was used to measure the fat content in permeate. The reducing sugar was investigated by using Lane and Eynon volumetric method (AOAC, 923.09), and formalin indices were assessed using standard methods (Institute of standards and industrial research of Iran; fruit juices, test methods; (No. 2685).

### 3.1 Formalin index

Briefly, for formalin index measurement, 25 ml of orange juice was titrated in a beaker with 0.25 N NaOH to pH 8.1 on the pH meter; then, 10 ml of neutralized formaldehyde solution was added. The solution was titrated potentiometrically to pH 8.1 with 0.25 N NaOH after about 1 min. The formalin index was calculated as follows:

$$F=\left\{\left(V\times N\times 10\right)/V_{0}\right\}\times 100$$

where V is the volume of the utilized NaOH, N is the normality of NaOH, and V_0_ is the volume of the sample.

### 3.2 Sensory evaluation

The sensory evaluation was performed by 10 trained panelists. Briefly, 60–70 ml of cool beverage was given to the panelists. Their location was constant during the sensory test, and the time of the sensory tests was selected around 11 a.m. The sensory testing was performed according to the linear method and the general acceptance of the beverage samples by the reviewers. Each time, the panelists were asked to mark the appropriate form after testing the drink, while considering the sensory properties such as color, taste, appearance, and sensation of the mouth. Between each assessment, the panelists consumed water to eliminate the taste of the previous sample. For linear evaluation, a linear length of 20 cm was drawn, and its two ends were defined as very desirable and very undesirable and the optimal term was placed in the middle. Each of the evaluators marked a linear line on this horizontal line after testing the samples that they were responsible for their evaluation. Finally, the sensory evaluation score was determined based on a related scale in which each 1 cm was considered a score (Sharma, Choudhary, Thakur, & Thakur, 2019).

### 3.3 Ascorbic acid measurement

The iodine titration test (iodometry) was used to measure vitamin C (Aghajanzadeh, Kashaninejad, & Ziaiifar, 2016). This method was used to determine the endpoint of titration from starch as a reagent. First, 20 ml of the orange drink was transferred to a 250 ml flask and mixed with 150 ml of distilled water. After adding 1 ml of 1% starch indicator solution, the solution was titrated with iodine solution until a blue color appeared.

### 3.4 Experimental design

The response surface methodology (RSM) was employed in two stages: (a) to study the effects of three qualitative factors (enzyme content, incubation temperature, and incubation time) and one nominal factor (heat treatment) in permeate lactose hydrolysis and (b) to evaluate the effect of the storage time (8 weeks) and the permeate amount (10%–40%) in the orange beverage prepared through using selected treatment from the first stage. After implementation, the variance and the regression analysis were performed on the data and the Fisher distribution was used to determine the significant effects (*p* < .05).

## 4 RESULTS AND DISCUSSION

### 4.1 Sensory evaluation

**FIGURE 2:**
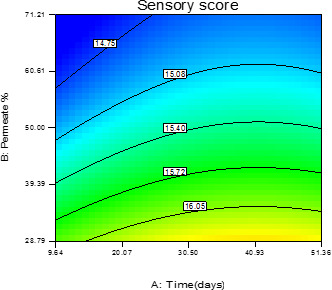
The interaction effect of storage days and permeate amount on sensory scores of beverage samples

The sensory properties of the orange beverage during 8 weeks storage at refrigerator temperature are shown in (Figure 2). As reflected by the coefficients in Table 3, the interaction effect of the storage time and the permeate amount on sensory evaluation of the beverages were significant (*p* < .05). With the increase of the permeate's percentage and by the predominance of its taste in the beverages, its acceptance by the evaluators was expected to decrease. According to the chart, samples with a higher percentage of permeate had a lower score, and samples with a lower percentage of permeate, even in a longer period of time, were able to receive a higher score in sensory evaluation. Findings of Sakhale, Pawar, and Ranveer (2012) on a whey‐based mango beverage showed that the reduction of existing water in mango juice beverage declines overall desirability score. As shown in Figure 2, the less overall desirability was reached by the increment of the amount of the permeate and the storage time, which is probably due to the production of the aromatic compounds caused by the chemical reactions in the beverage. Therefore, the highest overall desirability was found for the samples with 35% of permeate at day 41.

**Table 3:** Regression coefficients of the second‐order polynomial model for the response variables

Factors	Responses						
	Vitamin C	Density	Sensory evaluation	Reducing sugar	Total solid	Brix	Formalin index
Constant	–	1.02925	15.388	6.260435	12.74	12.30175	4.442213
A	–	–	0.200171	–	–	–	–
B	−0.15934	−0.01398	−0.74784	0.472236	−0.5217	−0.00445	−1.16447
AB	–	–	–	–	–	–	–
A × A	–	–	−0.19213	–	–	–	–
B × B	0.044	–	0.182875	−0.09696	0.645	0.182112	1.150043
*R* ^2^	.973067	.919594	.940481	.973431	.936837	.909329	.974564
Adjusted *R* ^2^	.967082	.911554	.910722	.968117	.924204	.88918	.968911
*F* value _Model_	162.5816	114.3696	31.60283	183.191	74.15966	45.12987	172.4135

Note

Factors in second stage: A: storage time; B: permeate amount.

### 4.2 Reducing sugar analysis

**FIGURE 3:**
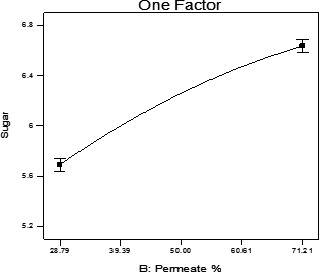
The effect of permeate amount on reducing sugar of beverage samples

The effect of the permeate amount on reducing sugar was significant (*p* < .05). As shown in Figure 3, increasing the permeate amount leads to an increasing trend of reducing sugar in the beverage, which is due to the hydrolyzed lactose in the permeate. On the other hand, another contributing factor that should be noted is the role of beverage citric acid on the sucrose hydrolysis and breaking it down into reducing sugars.

In a research on lemon beverage based on the hydrolyzed whey by Singh et al. (2014), a significant positive association was seen between the amount of the hydrolyzed whey and the sugar in the beverage. Bayoumi et al. (2011) observed that with the increase of the permeate percentage, the amount of reducing sugar in chocolate milk increased, while the required amount of sugar decreased from 7% to 5%.

### 4.3 pH changes

**FIGURE 4:**
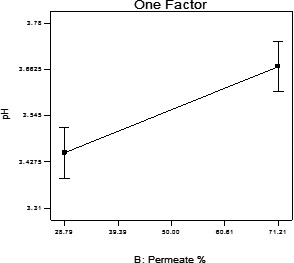
Changing trend of PH in accordance with permeate percentage

A significant positive relationship was detected between permeate amount and beverage's pH (*p* < .05), which is in line with those reported by Beucler et al. (2005). They observed that the beverage's pH had an increasing trend and the beverage with a total of 100% permeate had the highest pH as 4.37 (Figure 4).

Naik, Khare, Choudhary, Goel, and Shrivastava (2009) reported similar results in a study on the watermelon beverage and Dhamsaniya and Varshney (2013) reported the same in a research on banana beverages prepared from the permeate.

### 4.4 Total solid changes

**FIGURE 5:**
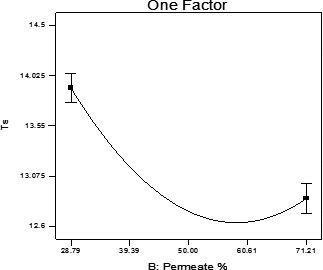
Total solid content of beverage samples through permeate increment

We found that the permeate amount had a significant effect on the total solid (*p* < .05). By increasing the proportion of the beverage's permeate, a decreasing trend was seen in TS amount (Figure 5). By increasing the permeate percentage, the amounts of sugar were decreased, and therefore, reducing the amount of TS was expected. Finally, it is expected that the slight increase of TS at higher amounts of the permeate is because of the increase of the permeate related salts and compounds in the beverage.

### 4.5 Density measurement and formalin index

The permeate percentage significantly declined the product density and the formalin index (*p* < .05). Indeed, by increasing the permeate percentage and decreasing the percentage of sugar in formulation of beverage, product density showed a downward trend.

**FIGURE 6:**
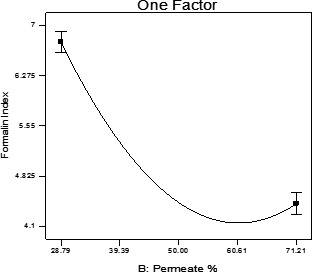
Formalin index of beverage samples through permeate increment

The amount of the formalin index has dropped, which could be due to the chemical reaction of the beverage's amino acids with other compounds, such as reducing sugars on the Maillard reaction (Kabasakalis, Sipidou, & Moshatou, 2000) (Figure 6).

### 4.6 Vitamin C analysis

**FIGURE 7:**
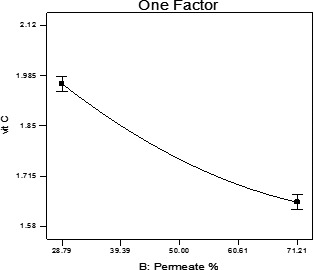
The effect of permeate amount on ascorbic acid amount of orange beverage samples

Our analysis indicated that the effect of the permeate percentage on the trend change of vitamin C was significant (*p* < .05). In addition, the high adjusted *R* ^2^ value (.99) was obtained for the corresponding predictive model (Table 3). As shown in Figure 7, the amount of the beverage's vitamin C was decreased. This may be due to the changes in the factors such as temperature, time, shelf life, and light that affect the amount of this vitamin. In a research conducted by Sakhale et al. (2012), on whey‐based Mango beverage, it was determined that the amount of vitamin C has decreased, which may be resulted from the auto‐oxidation and the exposure to the light during the storage in the refrigerator.

Exposure to the light, the storage duration, and the oxygen are the important factors affecting the vitamin C amount in the citrus. The increase of the storage temperature is also another degrading factor, and it has been reported that pasteurization reduced the amount of ascorbic acid up to 2%–6% (Das, Bhattacharjee, & Bhattacharjee, 2013; Torregrosa, Esteve, Frigola, & Cortes, 2006).

## 5 CONCLUSION

The orange beverage prepared from the permeate not only has the higher nutritional value compared to other available fruit beverages but also it can compete with other beverages, considering its organoleptic properties. The production of this beverage is a good way to reduce the environmental pollution caused by the permeate waste, to recycle a high nutritional value material and to produce a low price product. Based on our findings, it can be concluded that the permeate orange beverage would have at least 2 months shelf life at the refrigeration temperature. Moreover, the optimum amount of using the permeate is calculated as 35% and the optimum storage period is considered as 41 days, in which all of the vitamin C‐related contents and the sensory evaluation showed high levels. Considering the results of the research, producing orange beverages by using the favorable permeate percentage is recommended.

## CONFLICT OF INTEREST

The authors declare no conflict of interest.

## ETHICAL APPROVAL

The human and animal testing was unnecessary in the current study.
